# Hormonal contraceptive use is associated with love intensity across 51 countries

**DOI:** 10.1038/s41598-026-56430-8

**Published:** 2026-06-16

**Authors:** Marta Kowal, Piotr Sorokowski, Michal Misiak, W. P. Malecki, Agnieszka Sorokowska, S. Craig Roberts

**Affiliations:** 1 IDN Being Human Lab – Institute of Psychology, University of Wrocław, Wrocław, Poland; 2 Institute of Psychology, University of Wrocław, Wrocław, Poland; 3 Institute of Polish Philology, University of Wrocław, Wrocław, Poland; 4 Division of Psychology, University of Stirling, Stirling, UK; 5 School of Anthropology & Museum Ethnography, University of Oxford, Oxford, UK

**Keywords:** Romantic love, oral contraceptives, hormonal birth control, cross-cultural, Triangular theory of Love, TLS-15, Endocrinology, Health care

## Abstract

Hormonal contraception has revolutionized women’s reproductive control, yet its effects on romantic relationships are underexplored. We tested the Congruency Hypothesis, which predicts that changes in hormonal contraceptive (HC) use after forming a romantic relationship can reduce relationship quality. Using semi-representative samples from 51 countries (*n* = 10,482), we analyzed partnered women aged 45 or younger (*n* = 2,224) who reported HC use both when they met their partner and at the time of the study, and who rated their relationship satisfaction and intensity of the three components of love: Intimacy, Passion, and Commitment toward their partner. We found no robust support for the Congruency Hypothesis. However, in a weighted model, participants who changed their HC use pattern reported slightly lower Passion than those who consistently used (or consistently did not use) HC. Our study contributes to the growing body of research on the role of HCs by offering some reassurance: for most women, choosing or changing HCs is unlikely to meaningfully alter their relationship quality, including their experience of love and relationship satisfaction. We acknowledge, however, that our study was underpowered to detect very small effect sizes (e.g., *r* = .04) reported in a recent meta-analysis, and should therefore not be read as a definitive contribution in the ongoing debate. Although such small effects are generally considered practically negligible at the population level, they may nonetheless carry personal relevance for individual women.

**Supplementary Information:**

The online version contains supplementary material available at https://doi.org/10.1038/s41598-026-56430-8.

## Introduction

Female hormonal contraception became commercially available in 1960. Within just five years, this form of birth control—initially referred to as *the pill*—became the most widely used contraceptive method in the United States^1^. Its popularity quickly spread worldwide. Today, it is estimated that millions of women across the globe use hormonal contraceptives (HCs;^2^, with even more having tried this method at least once in their lifetime^3,4^. The primary function of HCs is to prevent pregnancy by suppressing gonadotropin release, thereby halting ovulation and inducing temporary infertility^5^. However, HCs may also exert numerous other physiological, psychological, and behavioral effects.

At a physiological level, HCs are associated with reduced natural fluctuations in progesterone and estrogen across the menstrual cycle^6^. Women who use HCs no longer experience natural cycles but remain in a state resembling the post-ovulatory luteal phase^6^. HC use has also been linked to decreased testosterone levels^7,8^, increased levels of sex hormone-binding globulin^9^, and disrupted oxytocin functioning^10^.

A growing body of evidence connects these endocrine changes to a range of psychological outcomes. For instance, HC use has been associated with severe mood changes^11^, decreased well-being^12^, and altered affective responsiveness^13^. Beyond these general psychological effects, HC use has also been linked to a variety of mating-relevant behaviors, including altered patterns of mating competition and intrasexual rivalry^14^, as well as reduced sexual desire and broader sexual dysfunction^15^. Of particular interest is their potential influence on romantic relationships, from early mate preferences to later relationship functioning. For instance, some studies indicate that HC use alters or diminishes naturally occurring preferences for the body odour of genetically dissimilar men, specifically at human leukocyte antigen (HLA) genes^16,17^, perhaps because of a hormonally mediated preference to be around kin during pregnancy and because the pattern of key reproductive hormone levels during HC use resembles those experienced during pregnancy^17,18^.

Another group of studies has focused on phenotypic facial or vocal cues, suggesting that HC users tend to show weaker preferences for the faces or voices of masculine men compared with naturally cycling women^19–21^, which may have meaningful implications for mating behavior and long-term partner choice^22^. One theoretical framework used to explain this second category of mate preference effect is the ovulatory shift hypothesis, according to which women’s mate preferences fluctuate across the ovulatory cycle. During the ovulatory (fertile) phase, women are said to exhibit stronger preferences for “cads”—men who are more masculine and physically attractive, and who presumably offer better genetic material for offspring. In contrast, during the post-ovulatory phase, female preferences shift toward “dads”—men who are more nurturing and dependable and more likely to invest in long-term care for both the woman and the child^23^. Unlike naturally cycling women, HC users are effectively stabilized in a state mimicking the post-fertile phase of the cycle^6^, which could account for their reduced preference for masculine traits in men^19,20^.

The ovulatory shift hypothesis has also been extended to examine dynamics within existing romantic relationships. Researchers have investigated whether naturally cycling women differ from HC-using women in terms of sexual satisfaction and overall relationship quality, with the prediction that HC users would report lower levels of both. However, findings have been mixed. While some studies support this prediction, others do not [for a review, see ].

Roberts et al.^25^ proposed an alternative hypothesis, suggesting that what matters more than HC use per se is the congruent usage patterns across time. According to this view, if women met their future partners while using hormonal contraception and later discontinued its use, they might perceive their partners as less appealing than they did while using hormonal contraception. Similarly, if women met their partners while not using HCs and later began using them, they might also perceive their partners as less compatible with them than before. Roberts et al.^25^ hypothesized that such changes in HC use would lead to lower relationship and sexual satisfaction. Testing this “Congruency Hypothesis” in a sample of 2,519 parous women, they found empirical support for their predictions. Further support came from subsequent cross-sectional^26,27^ and longitudinal studies^28,29^. However, more recent studies (some with very high power) have failed to replicate these effects^29–32^.

This mixed evidence means that further studies are required to ascertain the potential effects of HC use during partner choice on women’s subsequent relationship quality. Furthermore, because all the above-described research has been conducted predominantly with WEIRD (Western, Educated, Industrialized, Rich, and Democratic;^33)^ populations, further work is especially required to investigate whether such effects might also be present in non-WEIRD societies.

To address this gap, we conducted a large-scale, cross-cultural investigation of the role that HC use may play in women’s romantic relationship quality. Specifically, we focused on two facets of relationship quality: relationship satisfaction^34^ and the intensity of love, a central pillar of romantic relationships^35^. We measured love as operationalized by Sternberg’s^36^ Triangular Theory of Love, which characterizes love by three core components: *Intimacy*, reflecting feelings of emotional closeness, mutual understanding, and connectedness; *Passion*, encompassing sexual attraction, physical desire, and romantic arousal; and *Commitment*, referring to the cognitive decision to maintain the relationship over time^36^.

In line with the Congruency Hypothesis, we predicted that partnered women who had changed their HC use since they met their current partner would report lower relationship quality compared to those whose usage patterns remained consistent (H1). Additionally, given that HCs influence several neurochemicals involved in romantic relationship experiences, including sex hormones and oxytocin^37^, we hypothesized that women who were using HCs at the time of the study would report lower overall levels of relationship satisfaction and love (H2). To test these hypotheses and enhance the generalizability of findings, we recruited semi-representative, quota-based samples from 51 countries. We controlled for key covariates, including age, relationship length, and socioeconomic status, and analyzed data from a subsample of partnered women aged 45 or less, who reported on their HC use both at the time they met their partners and at the time of the study.

## Results

Participants (*n* = 2,224) were classified into four HC use groups: used HC in the past (specifically, when meeting their romantic partners) and at the time of the study (*n* = 395, 17.76%), used HC in the past but not at the time of the study (*n* = 241, 10.84%), did not use HC in the past but did use HC at the time of the study (*n* = 372, 16.73%), and used HC neither in the past nor at the time of the study (*n* = 1,216, 54.68%). Overall, 1,611 (72.44%) women had congruent HC-use status across the two time points, while 613 (27.56%) changed their HC-use status. Table S2 in the SM presents the bivariate correlations among the variables of interest. Figures S1–S4 present the distribution of participants who used HCs in the past, in the present, changed their patterns of HC use, and currently use any form of contraception across the countries.

The results of the generalized linear models revealed no significant links between the likelihood of using HCs at the time of the study and participants’ age, relationship length, and SES (all *p*s > 0.05). However, the analysis predicting congruent HC use revealed that women in longer (vs. shorter) relationships were more likely to change their HC use (log odds = − 0.046, *SE* = 0.008, 95%CI [− 0.063, − 0.029], *p* < .001). This finding corresponds to a ~ 4.5% decrease in the odds of reporting non-congruent HC use for each one-year increase in relationship length. There were no significant links between the pattern of HC use and age or SES.

The results of the linear mixed-effects models showed almost identical patterns whether or not covariates were controlled for. The only exception was that Intimacy was predicted by congruent HC use when not controlling for covariates; this is likely because congruent HC use was significantly related to relationship length (positively to past HC use and negatively to current HC use; see Table S2 in the SM). Therefore, here we present the results of models accounting for covariates (for detailed results with and without covariates, see Tables S3–S4 in the SM).

**Fig. 1:**
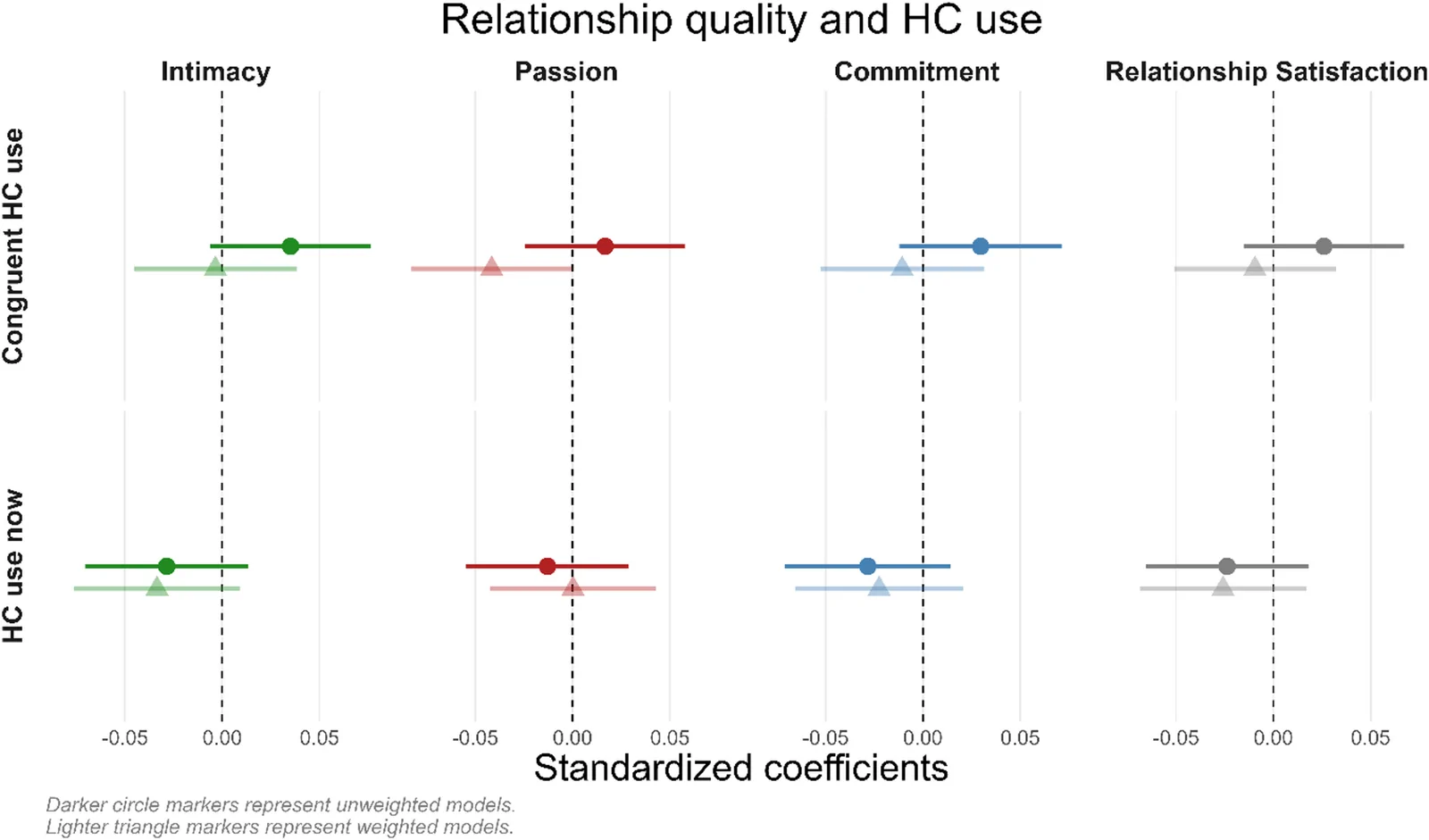
Four measures of relationship quality and patterns of HC use. Panel shows standardized coefficients for HC use congruence and current hormonal contraception use for the intensity of romantic love components (i.e., Intimacy, Passion, and Commitment) and relationship satisfaction, based on the results of the multilevel models, controlling for covariates (i.e., age, relationship length, and SES). Lighter color represents coefficients weighted with propensity scores. *Note* positive values represent higher relationship quality (i.e., love and relationship satisfaction) for those with a congruent pattern of HC use (Congruent HC use), while negative values represent lower relationship quality (i.e., love and relationship satisfaction) for those currently using hormonal contraception (HC use now). Darker circle markers represent non-weighted coefficients, while lighter triangle markers represent weighted coefficients.

As shown in Fig. 1 (upper panel, darker circles), models controlling for covariates but not weighted with propensity scores did not provide evidence for significant links between congruent HC use and any of the relationship quality outcome variables. Weighted models with propensity scores (lighter triangles) yielded largely similar results, except for Passion, which was marginally negatively related to congruent use (*p* = .049), suggesting that those who changed their pattern of HC use reported slightly lower levels of Passion (β = −0.042, SE = 0.021, 95%CI[− 0.083,0.000]). For the current HC use, both unweighted (darker circles) and weighted (lighter triangles) models did not provide evidence for significant links between HC use and Intimacy, Passion, Commitment, and Relationship Satisfaction (lower panel; Fig. 1; see also Table S5 in the SM).

**Fig. 2:**
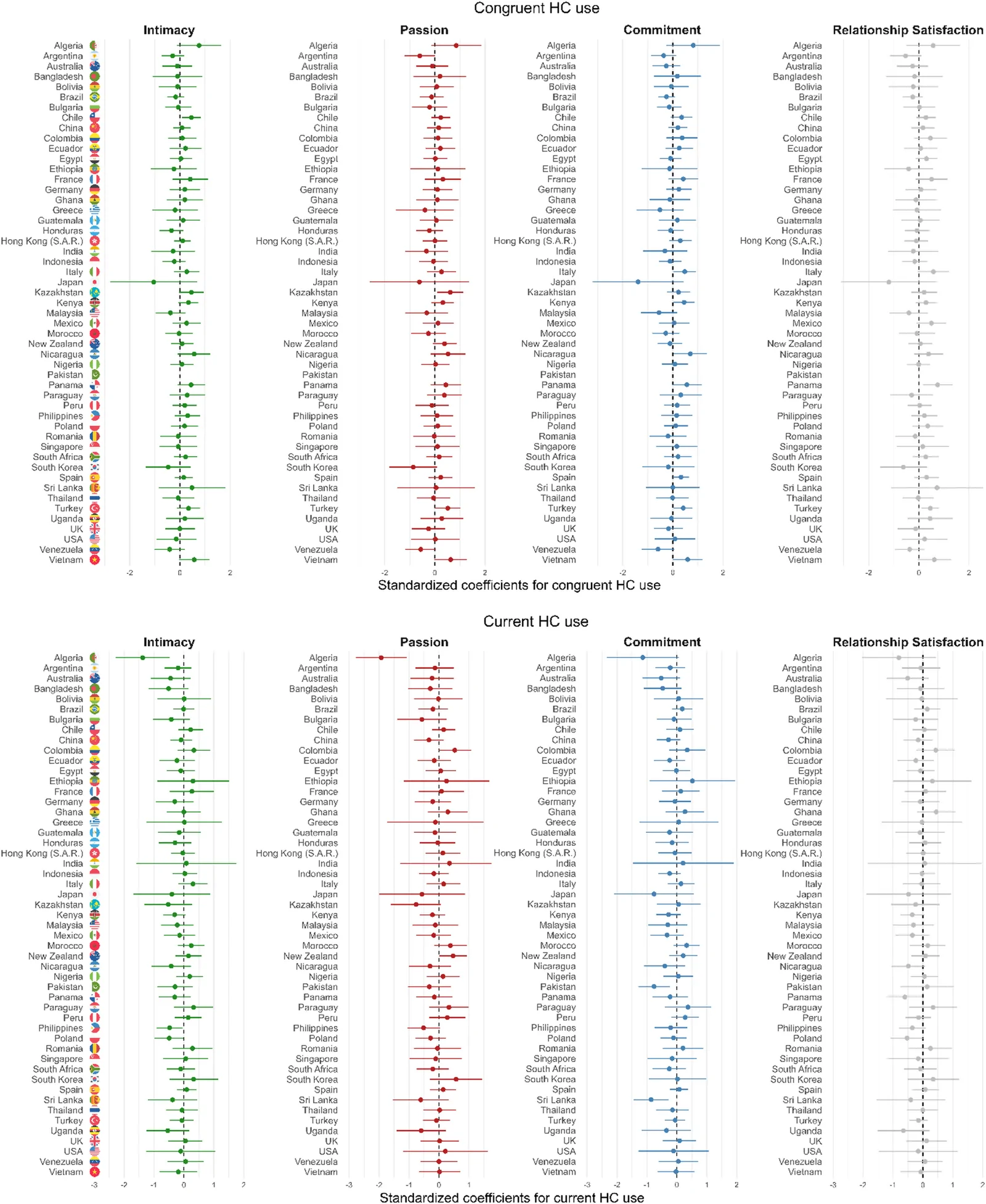
Standardized coefficients for HC use congruence and current HC use for the intensity of romantic love components (i.e., Intimacy, Passion, and Commitment) and relationship satisfaction across countries, controlling for covariates (i.e., age, relationship length, and SES). *Note *positive values represent higher relationship quality (i.e., love and relationship satisfaction) for those with a congruent pattern of HC use (Congruent HC use), while negative values represent lower relationship quality (i.e., love and relationship satisfaction) for those currently using HCs. Sample sizes across countries ranged from 20 to 77 (see Table S1 in the SM for details).

Standardized coefficients for congruent HC use and current HC use substantially varied across countries (see Fig. 2). The interactions between past and present HC use for all four variables of interest were non-significant. Figure S5 in the SM presents the weighted means of love components and relationship satisfaction across the four groups: HC use in both the past (when meeting a romantic partner) and the present (at the time of the study), HC use in the past but not in the present, HC use in the present but not in the past, and HC non-use in both the past and the present).

We conducted two additional sensitivity analyses to ensure the robustness of our findings. First, we repeated all analyses on a subsample of exclusively heterosexual women (*n* = 1,808), which yielded results consistent with the full-sample analyses (see Table S6 in the SM). Second, given the notably wide confidence intervals observed for Japan and India in Fig. 2 (suggesting these countries may exert disproportionate influence on the overall results), we reran all analyses excluding these two countries. This sensitivity analysis likewise yielded no significant links between congruent or current HC use and any of the four relationship quality outcomes (see Table S7 in the SM), confirming that the overall pattern of results is not driven by these specific countries. Finally, a variable that estimates the cultural distance of each country from the USA as a measure of its “WEIRDness” (taken from Muthukrishna et al.^38)^ did not moderate the links between congruent HC use, current HC use, and relationship quality (see Table S8 in the SM; note that this analysis was restricted to the 39 countries for which the index was available).

## Discussion

In our large-scale, cross-cultural study spanning 51 countries, we sought to disentangle the relationship between HC use and experiences of love and relationship satisfaction. Unsurprisingly, women in longer relationships were more likely to report a non-consistent pattern of HC use. Analyzing data from 2,224 partnered women aged 45 or younger and controlling for age, relationship length, and SES, we found no robust differences in relationship quality between those who consistently used HCs and those who did not, either at the time they met their partners or at the time of the study. The only exception was Passion, which was marginally significantly associated with the pattern of HC use (congruent or non-congruent). On average, women who changed their HC use (i.e., either using HC in the past but not at the time of the study or not using HC in the past but using it at the time of the study) reported 1.6% lower average Passion than those who had not changed their HC use (i.e., either consistently using or not using HC). Importantly, this effect emerged only in the propensity-scored analysis and was not apparent in unweighted models. Furthermore, there was substantial variability across countries, but our moderation analysis did not reveal evidence that “WEIRDness” shapes the links between HC use and relationship quality. However, the indirect nature of the “WEIRDness” measure and the limited per-country sample sizes mean that firm conclusions about cross-cultural generalizability remain premature.

Contrary to the Congruency Hypothesis, we found no evidence that consistency of HC use—either using or not using HCs—was linked to differences in relationship quality, as measured with Intimacy, Commitment, and Relationship Satisfaction. Partnered women who maintained a consistent pattern of HC use reported levels of relationship quality that were similar to those who changed their use patterns. While early studies supported congruency effects on relationship outcomes^25–27^, our findings align with more recent, well-powered research showing no associations between congruent HC use and sexual or relationship satisfaction^30,32^. Importantly, our study extends this work by focusing not only on relationship satisfaction but also on the experience of love.

Love is a central aspect of romantic relationships, motivating their initiation, maintenance, and, when it fades, dissolution^35,39,40^. It is typically divided into passionate and companionate subtypes^41^. Passionate love often peaks early in relationships^42^ and may decline over time, leading either to breakups or to the development of companionate love—characterized by deep attachment and emotional bonds that sustain long-term partnerships^43,44^.

Even if initial attraction levels fluctuate—whether for other reasons or in conjunction with HC use—love, especially its companionate form, may follow a relatively universal trajectory that is resilient to minor changes in perceived partner appeal^45^. This resilience could explain the lack of robust differences in love intensity between women with consistent and inconsistent patterns of HC use. These results add to recent findings of no apparent differences in love intensity between arranged and free-choice marriages^46^, suggesting that love can develop and flourish regardless of initial attraction or perceived compatibility.

However, love is also thought to have biological underpinnings^47^. Numerous physiological pathways have been implicated in the experience of love [for reviews, see ], including oxytocin, which appears to interact with HC use^7,8,10^. Often dubbed “the love hormone”, oxytocin plays a central role in human social bonding and, in particular, pairbond formation between romantic partners^49^. Disruptions to oxytocin signaling may impair attachment and thus deteriorate relationship quality. Indeed, a functional magnetic resonance imaging study found that the typical reward-region oxytocin response to one’s partner’s face was blunted in women using HCs^50^. Interestingly, our study did not provide evidence of any significant links between HC use and Intimacy, Commitment, and Relationship Satisfaction.

The small, borderline-significant effect of congruent HC use on Passion (*p* = .049) warrants particularly cautious interpretation. Although statistically significant in the propensity-score weighted model, this association was not apparent in the unweighted analyses. This non-robust pattern suggests confounding rather than a direct pharmacological effect of HC on passionate love. A plausible confounding pathway may involve relationship duration: bivariate correlations indicated that changes in HC use patterns were more common among women in longer romantic relationships. Given that passionate love most commonly declines with increasing relationship length^43,51^, women in longer romantic relationships would be expected to report lower Passion overall, independently of any HC-related effects. This overlap between relationship duration and non-congruent HC use may have resulted in a spurious negative association between changing HC use and Passion, particularly in the weighted model, which attempts to adjust for group composition but which cannot fully disentangle temporally confounded variables.

These findings may appear especially surprising, given the substantial literature documenting that HC use is associated with reduced sexual desire and altered sexual function^15,52^. Several non-mutually exclusive explanations may account for these results. First, the Passion subscale of the TLS-15 measures romantic passion within the relationship broadly, capturing feelings of attraction, romantic arousal, and personal desire for ’one’s partner, rather than sexual desire or sexual function per se^36^. These are conceptually and empirically distinct constructs, and it is plausible that HC-related reductions in sexual desire, which are more consistently documented in experimental designs^52^, do not translate straightforwardly into diminished romantic passion within an established relationship. Romantic passion in longer-term partnerships may be sustained by factors beyond hormonal sexual drive, including shared identity, emotional investment, and relationship history^43^. Second, the biological pathway most plausibly linking HC use to changes in Intimacy, disrupted oxytocin signaling and its effects on social bonding^50^, is mechanistically distinct from the androgen pathway governing sexual desire. HC use has been found to reduce free testosterone levels^8^, which may decrease libido without substantially altering the broader emotional experience of romantic passion. Third, as discussed in the limitations section, our study may have been underpowered to reliably detect small links between Passion and congruent HC use. Consistent with this possibility, the bivariate correlation between the two variables was very small (*r* = .03), suggesting that a true association of very small magnitude cannot be fully ruled out.

Finally, we observed substantial cross-national heterogeneity in HC use. Current HC use ranged from approximately 2.9% in India to 71.1% in Honduras, a range that almost certainly reflects differences in HC access, healthcare infrastructure, regulatory history, and cultural attitudes toward hormonal methods rather than differences in reproductive preferences alone. The very low rates of HC use observed across several Asian countries in our sample, including India, Japan, and Kazakhstan, are consistent with the historically late approval of oral contraceptives in Japan and with documented preferences for non-hormonal methods such as condoms^53^. Conversely, higher rates of HC use in countries such as Morocco, Chile, and Egypt may reflect specific healthcare policies or patterns of gynecological care that facilitate access to hormonal methods^54^. Beyond access, cultural dimensions known to shape relationship dynamics, such as individualism-collectivism and gender equality, may also moderate the relationship between HC use and relationship quality^45,51^. However, because our per-country sample sizes (ranging from 20 to 77) preclude formal within-country analyses, these interpretations remain speculative.

We did, however, conduct one moderation analysis focusing on countries’ WEIRDness [operationalized as cultural distance from the USA; 38]. The results did not provide evidence for any significant moderation of the links between HC use and relationship quality by countries’ WEIRDness, suggesting that the null associations observed in our data are not specific to Western or highly modernized contexts. However, these results warrant caution before drawing any firm conclusions. The Muthukrishna et al.^38^ index captures cultural distance from the USA as a proxy for WEIRDness, which is necessarily an indirect and imperfect operationalization. More fundamentally, the per-country sample sizes in our study (ranging from 20 to 77) preclude formal within-country analyses or reliable subgroup comparisons between WEIRD and non-WEIRD countries. Thus, the moderation analysis we report reflects population-averaged associations across countries varying in WEIRDness, rather than a direct test of the hypothesis within non-WEIRD populations. Future studies collecting larger within-country samples (especially from underrepresented non-WEIRD regions) will be essential for addressing this question more rigorously.

## Limitations and future directions

Our study is not without limitations. First, randomized placebo-controlled trials and meta-analyses have reported that HCs can diminish sexual desire^15,52^, whereas large cross-sectional studies have linked their use to increased sexual frequency^30^. These apparently conflicting findings may reflect selection effects—women with stronger baseline sexual interest may preferentially choose HC methods. Similarly, observed associations (or the lack thereof) may reflect reverse causality or stable selection processes rather than causal effects^55^. For instance, pre-existing relationship closeness or stability might influence the decision to use HC, rather than HC use shaping experienced intimacy. Likewise, women dissatisfied with HC side effects may discontinue use or experience relationship dissolution—either because of other factors or—what we deem rather unlikely based on the prior evidence^30,32^—because of the negative impact of HC use on their relationship quality, including love experiences and relationship satisfaction. These examples highlight the need to interpret correlational data cautiously.

Another limitation of our study concerns its cross-sectional, between-person design. As French and Meltzer^56^ have argued, such designs are particularly vulnerable to selection effects, as women who choose to use, discontinue, or change hormonal contraception may differ systematically from one another in ways that also predict relationship quality, independent of any pharmacological effect of HC use. This concern is reinforced by the meta-analytic evidence of Shiramizu et al.^29^, who found no reliable effects at the between-person level, while identifying weak but statistically significant effects at the within-person level. Within-person designs, in which the same individuals are tracked across changes in HC use, would therefore offer a more rigorous test of causal hypotheses^56^, and we encourage future researchers to employ such longitudinal designs with large, diverse samples.

Additionally, our sensitivity analysis indicated that while we had sufficient power to detect the effect size reported in the original study proposing the Congruency Hypothesis (*r* = .13;^27^, we lacked sufficient power to reliably detect the very small effect size (*r* = .04) reported in Shiramizu et al.’s meta-analysis^29^. Our study should therefore not be interpreted as a definitive, high-powered test of the Congruency Hypothesis, and the absence of significant effects in our analyses cannot be taken as definitive evidence of absence. We therefore cannot rule out the existence of such small effects, and we encourage future researchers to design studies specifically powered to detect them. Furthermore, interpreting what effect size is practically meaningful in this context is not straightforward. For instance, some scholars^57^ have argued that even small effects, such as *r* = .05, can be of real-world relevance depending on the outcome in question, and relationship quality is arguably one such outcome. It is therefore possible that a population-level effect of *r* = .04, though small by conventional benchmarks, may nonetheless be subjectively meaningful for some women. Our findings should thus be interpreted cautiously: they do not provide evidence for a meaningful effect of HC use on love or relationship satisfaction at the population level, but they equally cannot be taken as definitive evidence of absence, particularly for very small effects or for individual women whose sensitivity to hormonal changes may exceed the population average.

A further limitation concerns the retrospective nature of our measure of past HC use. Although participants were provided with an “*I can’t remember*” response option, only 53 women (1.6% of the full sample) selected it, and these individuals were aged over 45 years and were therefore excluded from the main analyses. While recall bias cannot be entirely excluded (especially for participants in longer relationships), the very low rate of reported uncertainty across the full sample suggests this is unlikely to be a substantial source of bias in the present study. Future research would nonetheless benefit from prospective or longitudinal designs that capture HC use at the time of relationship formation. Moreover, although our samples spanned 51 countries with predefined quotas for age, gender, and residential area, they did not capture the full breadth of global diversity, nor were they nationally representative across all sociodemographic respects. Because per-country sample sizes varied (from 20 to 77), we refrained from detailed within-country analyses. Future studies may collect larger samples to provide more fine-grained analyses of cultural variability in the links between relationship quality and HC use, as well as the moderating role of socio-cultural factors.

Finally, a potential concern regarding statistical power is the imbalance in group sizes between women with consistent (72%) and inconsistent (28%) patterns of HC use. We acknowledge that while a 50–50 distribution is mathematically optimal for maximizing statistical power, it is an arbitrary one; the distribution we used reflects the ecological prevalence of HC use patterns in the populations we studied and was a natural consequence of prioritizing external validity and representativeness. Quantitatively, using the effective sample size formula^58^, a 72 − 28% split retains approximately 80.6% of the statistical power of a perfectly balanced design with the same total sample size. Methodological research indicates that imbalance ratios exceeding 1:4 may be a source of concern^59^, whereas our ratio of approximately 2.5:1 falls well within the range considered acceptable for observational research.

## Conclusions

Misinformation about hormonal contraception and its psychosocial side effects remains widespread^60,61^, and our findings may help offer some limited reassurance in this context. Before drawing on our results, however, an important caveat must be acknowledged: our study was underpowered to detect the very small effect sizes (*r* = .04) reported in the most recent meta-analysis^29^, and should not be read as a definitive argument in the ongoing debate. With this limitation in mind, we note that we observed no robust differences in relationship quality between women who changed their HC use patterns and those who did not after meeting their partners, nor between current HC users and non-users. We observed only one borderline-significant effect of congruent HC use on Passion; however, it was very small in magnitude and varied considerably across individuals and countries. Although caution is warranted and further studies are needed, our results may offer some reassurance to women considering whether to initiate or change their use of hormonal contraception.

## Method

This study was not preregistered. All data, study materials, and analysis scripts are publicly available at https://osf.io/fh9t5/overview? view_only=ad769770053e418f9b4f265e5a6e75d7. The Institutional Review Board (IRB) at the Institute of Psychology, University of Wrocław, approved the study protocol. All participants provided informed consent before participating in the survey, in accordance with the Declaration of Helsinki, and were compensated in accordance with the survey company’s standard practices.

## Participants

This study was part of a large cross-cultural project on romantic relationships^51,62^. In this study, we aimed to recruit semi-representative samples from 51 countries and areas of at least 200 participants per country, with quotas applied for gender (~ 50% women and ~ 50% men), age (25% aged 18–27, 25% aged 28–37, 25% aged 38–47, and 25% aged 48 or older), and residential area (urban vs. rural, as classified by CIA data, 2024). Participants were recruited via Syno International, an external survey company using online panels. While these quotas were designed to improve sample diversity within each country, they do not ensure full national representativeness across all sociodemographic dimensions, including education, income, or healthcare access. For simplicity, we refer to all participating locations as “countries,” although one of them, Hong Kong, is a Special Administrative Region of China rather than a sovereign state.

**Fig. 3:**
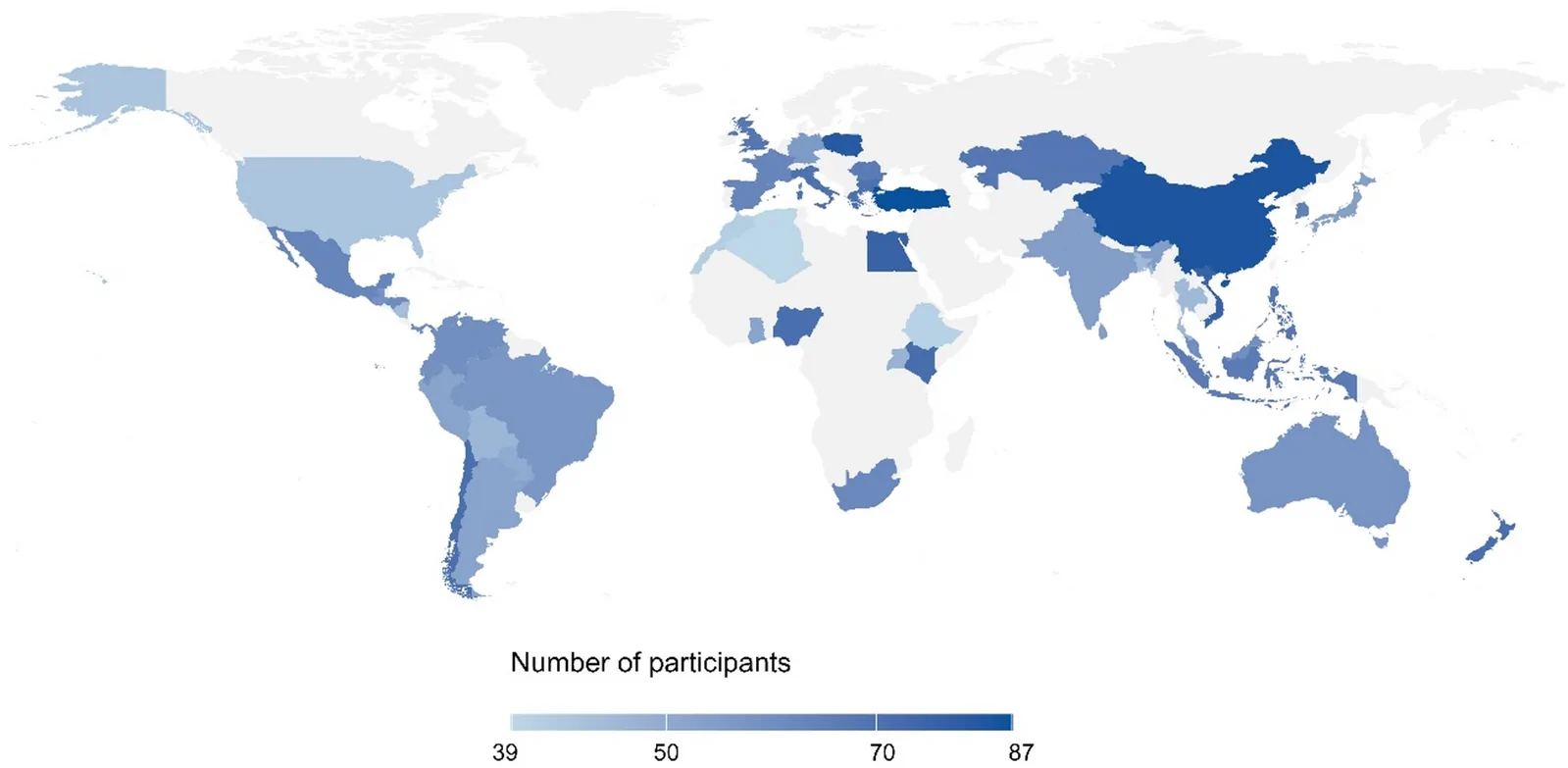
A distribution of the participants (*N* = 3,082) across participating countries and areas (*n* = 51). *Note* Gray areas represent countries where we did not collect data.

This approach resulted in a total sample of 10,482 individuals, of whom 3,082 were women in romantic relationships at the time of the study (see Fig. 3). Our primary analytical sample consisted of partnered women aged 45 or younger, as women at or below this age are unlikely to experience menopause^63^ and therefore constitute the relevant population for studying active HC use. This resulted in a sample of 2,224 women with an average age of 32.26 (*SD* = 8.04, *Median* = 33) and a mean relationship length of 8 years (*SD* = 6.91, *Median* = 6). Most were married (*n* = 1,267, 57%), while 501 (23%) were in committed relationships and cohabiting, 441 (20%) were in committed relationships but not cohabiting, and 15 (1%) indicated the “Other” option when asked about their relationship status. Participants’ descriptive statistics across countries are presented in Table S1 in the Supplementary Materials (SM).

To determine the smallest effect size detectable with our sample size (*n* = 2,224) at 80% power, we conducted a simulation-based sensitivity analysis. Following the approach by Riesthuis^64^, we calculated the proportion of 1,000 simulations in which the 95% confidence interval successfully excluded zero. The analysis indicated that we had 80% power to detect a standardized coefficient of β = 0.06. Consequently, while our study was sufficiently powered to detect the effect size reported in the initial study proposing the Congruency Hypothesis (*r* = .13;^27^, we lacked sufficient power to reliably detect the smaller between-subjects effect found in a recent meta-analysis (*r* = .04;^29^.

### Measures

Participants provided basic demographic information via single-item questions: “*What is your age in years?*”; “*What is your gender?*”; “*What is your sexual orientation?*”; “*What is your current romantic relationship status?*“; and “*How long have you been in your current romantic relationship?*”. Socioeconomic status (SES) was assessed using the MacArthur Scale of Subjective Social Status, a 10-point ladder measure in which higher rungs indicate greater perceived SES^65^. Use of HCs was assessed with two items: “*Which forms of contraceptive do you currently use?*” with five response options, including “*None*”, “*Contraceptive pill*”, “*Another hormonal contraceptive*, e.g.,* implant*,* injection*”, “*Condom*”, and “*Other (please state)*”; and “*When you began your relationship with your current partner*,* which forms of contraceptive did you use?*” which included the same five options plus “*I can’t remember*.”

The intensity of experienced love was measured using the short version of the Triangular Love Scale (TLS-15), which had been forward- and back translated^66^ by professional translators. The scale’s reliability, validity, and measurement invariance across different language versions have been extensively tested^67^, including with the data used in this study^51^. The TLS-15 consists of 15 items, with five items assessing each of the three love components: Intimacy (Cronbach’s α = 0.92, McDonald’s ω = 0.93; exemplary item: “*I feel that my partner really understands me*”), Passion (α = 0.90, ω = 0.91; e.g., “*My relationship with my partner is passionate*”), and Commitment (α = 0.91, ω = 0.92; e.g., “*I view my relationship with my partner as permanent*”). Responses were recorded on a 5-point scale ranging from 1 “*Not at all*” to 5 “*Extremely*.” Relationship satisfaction was measured with a single-item question “*How satisfied are you with your relationship with your partner?*”, answered on a 5-point scale from 1 “*Not at all*” to “*Extremely*”^34^.

## Procedure

Participants were recruited via an external survey company using online panels in all 51 countries and areas. Data collection was conducted online, and participants were compensated in accordance with the company’s standard compensation practices.

### Statistical analysis

We employed a five-step plan on the full dataset to detect potentially careless responses, which are a common issue in online studies^68^. First, the survey company delivered data only from participants who passed all the attention checks. Second, we flagged data from participants who completed the survey in less than 10 min (*n* = 8). Third, the longest string of repetitive responses was calculated (e.g., answering 1,1,1,1,1,1,1,1,1,1 or 5,5,5,5,5,5,5,5 throughout the survey). Participants who reached the threshold for the maximum number of repetitive responses minus one standard deviation would have been flagged; however, no partnered women in the sample exceeded the threshold. Fourth, we calculated the intra-individual variability of responses. We set the threshold to the maximum value of intra-individual variability minus one standard deviation; this flagged 15 participants with unexpectedly high variability (e.g., who answered 1,2,1,2,1,2,1,2,1 or 1,5,1,5,1,5,1,5,1,5, etc.). Fifth, we flagged potential outliers (*n* = 247) using the Mahalanobis Distance with the usually recommended cutoff (i.e., < 0.001;^69,70^. Based on these criteria, data from 268 participants were flagged as potentially inattentive participants. However, since the pattern of results was consistent whether data flagged as potential careless responses were excluded or included, we retained all data for subsequent analyses.

Next, we computed a set of three binary variables. The first was a variable describing the participant’s HC use when meeting their current romantic partner, with 0 denoting non-hormonal contraception or no contraception (i.e., answers “*Condom*” or “*None*”), and 1 denoting HC use (i.e., “*Contraceptive pill*” or “*Another hormonal contraceptive*,* e.g. implant*,* injection*”); the answers “*Other*” (*n* = 125, 3.8% in the current HC use and *n* = 45, 1.4% in the past HC use) and “*I can’t remember*” (*n* = 53, 1.6%) were coded as missing data. The second was current HC use, with the same rule as above. The third was the Congruency variable, with 0 referring to non-congruent HC use (a woman used HCs when she met her romantic partner and now does not, or one who did not use HCs when meeting her partner and now does) and 1 referring to congruent HC use (i.e., a woman who used HCs when meeting her romantic partner and still does at the time of the survey, or a non-user when she met her partner who is still a non-user).

We then calculated participants’ mean scores on the TLS-15 (including Intimacy, Passion, and Commitment) and examined bivariate correlations between the variables of interest. Next, we examined the percentage of participants who used HCs in the past, in the present (both hormonal and any form of contraception), and those who were non-congruent, across all the countries.

In the first step of the main analyses, we conducted generalized linear mixed-effects models with two binary outcome variables: congruent HC use (coded as 0–Non-congruent, 1–Congruent) and current hormonal contraception use (coded as 0–currently not using HCs, 1–currently using HCs) regressed on participants’ age, relationship length, and SES. Participants were nested within countries.

In the second step, we employed linear mixed-effects models using maximum likelihood estimation. Again, we nested participants within countries. Love subscales and relationship satisfaction were regressed onto the binary congruent HC use variable (coded as 0–Non-congruent, 1–Congruent). In the next set of models, we accounted for participants’ age, relationship length, and SES. Subsequently, we investigated interactions between congruent HC use and each control variable. To check for collinearity, we computed the Variance Inflation Factor (VIF), using the guideline of VIF > 5 as an indicator of potential multicollinearity concerns^71,72^. Next, we repeated these analyses, substituting the congruent HC use variable with a variable reflecting current HC use (coded as 0–currently not using HCs, 1–currently using HCs). To better understand the country-level differences in the pattern of HC use, we also ran random-slope models. To address previous suggestions that the largest differences in relationship quality can be observed among consistent HC users and consistent non-users^32^, we also ran models with two HC use factors included (i.e., past and present) and their interaction.

Additionally, we considered the possibility of selection bias arising from the possibility that women with different HC usage patterns might have been more inclined to end their romantic relationships. Such an effect might have led to an overrepresentation of particularly satisfied women who had altered their HC use, thereby artificially diminishing the apparent differences in relationship satisfaction and love intensity between women using hormonal contraception congruently and non-congruently. To address this issue, we calculated the proportion of participants across relationship durations for both the congruent and non-congruent groups, and then performed propensity score matching. Specifically, we estimated a propensity score for each participant, defined as the conditional probability of being in the non-congruent (or congruent) group given the covariates of age, SES, and relationship length^73^. These scores were generated using logistic regression. We then applied full matching to balance the groups while retaining all participants^74^. This approach ensured that individuals with comparable propensity scores were appropriately weighted, reducing bias due to differences in group sizes and covariate distributions. We repeated step two using the adjusted weights from the propensity score analysis.

Next, we repeated the analyses in two restricted samples: (1) a subsample of heterosexual women and (2) a subsample excluding participants from the two countries with the widest 95% confidence intervals around the standardized coefficients (i.e., Japan and India). Finally, we conducted additional analyses to test whether the associations between HC use and relationship quality are moderated by a country’s “WEIRDness”^33^. To this end, we introduced an interaction term between congruent HC use and current HC use and a measure of cultural distance between each country and the United States (one of the WEIRDest countries), based on the index proposed by Muthukrishna et al.^38^. These analyses were restricted to the subset of countries for which this measure was available (i.e., 39 countries). All statistical analyses were conducted in R (version 4.4.1).

All analysis scripts are publicly available (https://osf.io/fh9t5/overview? view_only=ad769770053e418f9b4f265e5a6e75d7). **Computational reproducibility**: The computational reproducibility of the results has been rechecked.

## Supplementary Information

Below is the link to the electronic supplementary material.


Supplementary Material 1


## Data Availability

All primary data are publicly available (https://osf.io/fh9t5/overview? view_only=ad769770053e418f9b4f265e5a6e75d7).
