# Supplementary material for: Hormonal contraceptive use is associated with love intensity across 51 countries
Source: Sci Rep. 2026 Jun 16;16:27475. doi: 10.1038/s41598-026-56430-8 (PMC13534682; doi:10.1038/s41598-026-56430-8)
Supplement: Supplementary file 1 [file 41598_2026_56430_MOESM1_ESM.docx]

# Supplementary Materials

**Hormonal contraceptive use is associated with love intensity across 51 countries**

Marta Kowal^1*^, Piotr Sorokowski^2^, Michał Misiak^2^, W. P. Malecki^3^, Agnieszka Sorokowska^2^, S. Craig Roberts^1,4*^

^1^IDN Being Human Lab – Institute of Psychology, University of Wrocław, Wrocław, Poland

^2^Institute of Psychology, University of Wrocław, Wrocław, Poland

^3^Institute of Polish Philology, University of Wrocław, Wrocław, Poland

^4^Division of Psychology, University of Stirling, Stirling, United Kingdom

Corresponding authors: Marta Kowal (marta7kowal@gmail.com) and S. Craig Roberts (stewartcraig.roberts@uwr.edu.pl)

**Table S1**

Detailed participants’ descriptives across countries.

|  |  |  |  | **Hormonal contraception use^a^** | | | **Relationship_status** | | |  |
| --- | --- | --- | --- | --- | --- | --- | --- | --- | --- | --- |
| **Country** | **N (%)** | **Mean age (SD)** | **Mean relationship length (SD)** | **HC use in the past (%)** | **Current HC use (%)** | **Non-congruent HC use** | **In a relationship and not cohabiting (%)** | **In a relationship and cohabiting (%)** | **Married (%)** | **Mean SES (SD)** |
| Algeria | 24 (1.1%) | 31.93 (8.94) | 8.49 (6.04) | 6 (25%) | 5 (20.8%) | 5 (0.2%) | 10 (41.7%) | 1 (4.2%) | 11 (45.8%) | 5.49 (1.45) |
| Argentina | 38 (1.7%) | 30.38 (8.01) | 3.8 (3.01) | 14 (36.8%) | 17 (44.7%) | 19 (0.9%) | 9 (23.7%) | 16 (42.1%) | 13 (34.2%) | 6 (1.47) |
| Australia | 47 (2.1%) | 33.27 (7.69) | 11.02 (7.84) | 28 (59.6%) | 12 (25.5%) | 22 (1%) | 5 (10.6%) | 12 (25.5%) | 30 (63.8%) | 6.35 (1.29) |
| Bangladesh | 29 (1.3%) | 30.88 (7.32) | 10.96 (7.99) | 4 (13.8%) | 4 (13.8%) | 2 (0.1%) | 5 (17.2%) | 3 (10.3%) | 21 (72.4%) | 4.96 (1.37) |
| Bolivia | 29 (1.3%) | 29.43 (7.32) | 19.4 (12.58) | 13 (44.8%) | 20 (69%) | 15 (0.7%) | 6 (20.7%) | 8 (27.6%) | 15 (51.7%) | 5.46 (2.05) |
| Brazil | 44 (2%) | 32.49 (6.55) | 8.95 (6.8) | 19 (43.2%) | 28 (63.6%) | 19 (0.9%) | 9 (20.5%) | 5 (11.4%) | 30 (68.2%) | 6.35 (1.78) |
| Bulgaria | 52 (2.3%) | 31.94 (8.32) | 5.17 (4.77) | 7 (13.5%) | 7 (13.5%) | 10 (0.4%) | 8 (15.4%) | 23 (44.2%) | 21 (40.4%) | 5.23 (1.72) |
| Chile | 53 (2.4%) | 29.88 (9.02) | 9.44 (7.33) | 29 (54.7%) | 35 (66%) | 22 (1%) | 22 (41.5%) | 23 (43.4%) | 8 (15.1%) | 5.77 (1.39) |
| China | 64 (2.9%) | 31.32 (7.54) | 8.24 (6.57) | 9 (14.1%) | 10 (15.6%) | 11 (0.5%) | 1 (1.6%) | 1 (1.6%) | 62 (96.9%) | 6.16 (1.15) |
| Colombia | 48 (2.2%) | 30.53 (8.23) | 11.42 (6.7) | 20 (41.7%) | 27 (56.2%) | 19 (0.9%) | 22 (45.8%) | 15 (31.2%) | 10 (20.8%) | 5.73 (2.19) |
| Ecuador | 41 (1.8%) | 32.73 (8.49) | 4.8 (3.09) | 12 (29.3%) | 19 (46.3%) | 17 (0.8%) | 9 (22%) | 14 (34.1%) | 18 (43.9%) | 6.24 (1.58) |
| Egypt | 53 (2.4%) | 29.92 (7.62) | 4.43 (3.37) | 21 (39.6%) | 31 (58.5%) | 18 (0.8%) | 8 (15.1%) | #N/D | 45 (84.9%) | 5.72 (1.57) |
| Ethiopia | 20 (0.9%) | 34.84 (8.23) | 8.36 (4.7) | 3 (15%) | 8 (40%) | 7 (0.3%) | 1 (5%) | 3 (15%) | 16 (80%) | 6.12 (1.71) |
| France | 43 (1.9%) | 30.87 (8.53) | 5.6 (3.55) | 23 (53.5%) | 20 (46.5%) | 19 (0.9%) | 15 (34.9%) | 5 (11.6%) | 23 (53.5%) | 5.96 (1.6) |
| Germany | 45 (2%) | 37.06 (6.73) | 5.91 (4.51) | 20 (44.4%) | 13 (28.9%) | 15 (0.7%) | 6 (13.3%) | 17 (37.8%) | 22 (48.9%) | 7.56 (2.25) |
| Ghana | 36 (1.6%) | 35.34 (9.03) | 9.91 (6.61) | 14 (38.9%) | 19 (52.8%) | 7 (0.3%) | 24 (66.7%) | 4 (11.1%) | 7 (19.4%) | 7.06 (1.92) |
| Greece | 51 (2.3%) | 32.41 (7.76) | 6.63 (6.46) | 4 (7.8%) | 2 (3.9%) | 4 (0.2%) | 6 (11.8%) | 9 (17.6%) | 36 (70.6%) | 5.78 (1.59) |
| Guatemala | 44 (2%) | 32.83 (7.72) | 4.46 (2.71) | 11 (25%) | 15 (34.1%) | 14 (0.6%) | 5 (11.4%) | 17 (38.6%) | 18 (40.9%) | 6.24 (1.54) |
| Honduras | 45 (2%) | 35.6 (6.79) | 7.6 (5.32) | 24 (53.3%) | 32 (71.1%) | 16 (0.7%) | 9 (20%) | 22 (48.9%) | 14 (31.1%) | 6.79 (1.38) |
| Hong Kong (S.A.R.) | 49 (2.2%) | 30.4 (9) | 14.72 (12.63) | 8 (16.3%) | 5 (10.2%) | 7 (0.3%) | 6 (12.2%) | 7 (14.3%) | 36 (73.5%) | 5.53 (1.53) |
| India | 34 (1.5%) | 30.74 (7.84) | 5.12 (4.87) | 3 (8.8%) | 1 (2.9%) | 4 (0.2%) | 2 (5.9%) | 1 (2.9%) | 31 (91.2%) | 6.5 (2.05) |
| Indonesia | 47 (2.1%) | 29.68 (8.28) | 6.27 (4.03) | 11 (23.4%) | 20 (42.6%) | 11 (0.5%) | 2 (4.3%) | 2 (4.3%) | 43 (91.5%) | 5.66 (1.36) |
| Italy | 54 (2.4%) | 32.91 (7.91) | 8.98 (5.56) | 14 (25.9%) | 19 (35.2%) | 17 (0.8%) | 11 (20.4%) | 20 (37%) | 23 (42.6%) | 6.05 (1.99) |
| Japan | 42 (1.9%) | 31.11 (8.66) | 7.91 (5.28) | 3 (7.1%) | 2 (4.8%) | 1 (0%) | 12 (28.6%) | 1 (2.4%) | 29 (69%) | 6.3 (1.55) |
| Kazakhstan | 55 (2.5%) | 34.72 (7.13) | 5.62 (4.36) | 9 (16.4%) | 4 (7.3%) | 11 (0.5%) | 10 (18.2%) | 11 (20%) | 34 (61.8%) | 6.38 (2.13) |
| Kenya | 46 (2.1%) | 28.73 (6.96) | 5.65 (4.68) | 10 (21.7%) | 26 (56.5%) | 20 (0.9%) | 6 (13%) | 13 (28.3%) | 27 (58.7%) | 6.78 (1.39) |
| Malaysia | 41 (1.8%) | 33.49 (8.2) | 6.1 (5.34) | 13 (31.7%) | 12 (29.3%) | 7 (0.3%) | 3 (7.3%) | 4 (9.8%) | 33 (80.5%) | 6.18 (1.6) |
| Mexico | 47 (2.1%) | 31.89 (7.36) | 7.38 (4.91) | 10 (21.3%) | 20 (42.6%) | 14 (0.6%) | 11 (23.4%) | 11 (23.4%) | 25 (53.2%) | 5.93 (1.19) |
| Morocco | 29 (1.3%) | 35.12 (7.49) | 4.28 (3.29) | 16 (55.2%) | 16 (55.2%) | 6 (0.3%) | 5 (17.2%) | #N/D | 24 (82.8%) | 5.31 (1.31) |
| New Zealand | 53 (2.4%) | 34.17 (7.69) | 7.64 (3.79) | 20 (37.7%) | 17 (32.1%) | 17 (0.8%) | 7 (13.2%) | 13 (24.5%) | 33 (62.3%) | 6.91 (1.38) |
| Nicaragua | 37 (1.7%) | 32.08 (8.08) | 9.8 (7.36) | 14 (37.8%) | 19 (51.4%) | 17 (0.8%) | 13 (35.1%) | 12 (32.4%) | 12 (32.4%) | 5.73 (1.42) |
| Nigeria | 45 (2%) | 31.5 (8.09) | 10.69 (7.86) | 11 (24.4%) | 21 (46.7%) | 14 (0.6%) | 10 (22.2%) | 3 (6.7%) | 32 (71.1%) | 5.7 (1.51) |
| Pakistan | 39 (1.8%) | 30.75 (7.66) | 6.94 (4.39) | 5 (12.8%) | 5 (12.8%) | 39 (1.8%) | 7 (17.9%) | 2 (5.1%) | 30 (76.9%) | 5.39 (1.86) |
| Panama | 44 (2%) | 33.93 (7.45) | 7.39 (5.61) | 19 (43.2%) | 20 (45.5%) | 19 (0.9%) | 10 (22.7%) | 9 (20.5%) | 25 (56.8%) | 7.15 (1.65) |
| Paraguay | 36 (1.6%) | 33.24 (7.07) | 6.78 (4.8) | 15 (41.7%) | 16 (44.4%) | 15 (0.7%) | 5 (13.9%) | 26 (72.2%) | 5 (13.9%) | 6.07 (1.78) |
| Peru | 40 (1.8%) | 32.11 (7.67) | 7.2 (5.21) | 11 (27.5%) | 17 (42.5%) | 16 (0.7%) | 22 (55%) | 9 (22.5%) | 9 (22.5%) | 5.64 (1.42) |
| Philippines | 42 (1.9%) | 32.19 (8.13) | 5.7 (4.1) | 9 (21.4%) | 12 (28.6%) | 7 (0.3%) | 9 (21.4%) | 16 (38.1%) | 15 (35.7%) | 5.54 (1.68) |
| Poland | 55 (2.5%) | 31.17 (8.38) | 4.72 (4.1) | 10 (18.2%) | 16 (29.1%) | 14 (0.6%) | 11 (20%) | 21 (38.2%) | 23 (41.8%) | 5.93 (2.15) |
| Romania | 51 (2.3%) | 36.47 (6.97) | 7.53 (6.76) | 9 (17.6%) | 8 (15.7%) | 7 (0.3%) | 13 (25.5%) | 8 (15.7%) | 30 (58.8%) | 7.43 (1.72) |
| Singapore | 35 (1.6%) | 33.5 (7.75) | 10.97 (8.96) | #N/D | 4 (11.4%) | 4 (0.2%) | 3 (8.6%) | 1 (2.9%) | 31 (88.6%) | 5.87 (2.13) |
| South Africa | 45 (2%) | 28.48 (8.63) | 10.71 (10.23) | 13 (28.9%) | 29 (64.4%) | 22 (1%) | 12 (26.7%) | 9 (20%) | 24 (53.3%) | 4.98 (1.6) |
| South Korea | 38 (1.7%) | 32.95 (8.33) | 6.67 (4.9) | 1 (2.6%) | 5 (13.2%) | 4 (0.2%) | 7 (18.4%) | 1 (2.6%) | 28 (73.7%) | 6.26 (1.95) |
| Spain | 51 (2.3%) | 35.7 (8.19) | 4.95 (2.65) | 17 (33.3%) | 20 (39.2%) | 13 (0.6%) | 16 (31.4%) | 20 (39.2%) | 15 (29.4%) | 6.45 (1.19) |
| Sri Lanka | 34 (1.5%) | 32.79 (7.91) | 8.82 (6.29) | 6 (17.6%) | 5 (14.7%) | 3 (0.1%) | 6 (17.6%) | 12 (35.3%) | 16 (47.1%) | 5.92 (1.48) |
| Thailand | 38 (1.7%) | 31.07 (8.1) | 7.51 (5.97) | 13 (34.2%) | 16 (42.1%) | 9 (0.4%) | 2 (5.3%) | 8 (21.1%) | 28 (73.7%) | 5.89 (1.3) |
| Turkey | 77 (3.5%) | 32.58 (8.81) | 3.71 (3.62) | 16 (20.8%) | 16 (20.8%) | 10 (0.4%) | #N/D | #N/D | 77 (100%) | 6.83 (1.46) |
| Uganda | 32 (1.4%) | 32.03 (7.1) | 10.51 (4.54) | 3 (9.4%) | 13 (40.6%) | 9 (0.4%) | 12 (37.5%) | 13 (40.6%) | 5 (15.6%) | 7.36 (1.44) |
| UK | 48 (2.2%) | 34.64 (8.19) | 3.89 (2.94) | 29 (60.4%) | 24 (50%) | 12 (0.5%) | 8 (16.7%) | 16 (33.3%) | 24 (50%) | 5.69 (1.75) |
| USA | 35 (1.6%) | 31.16 (7.88) | 7.33 (6.94) | 10 (28.6%) | 3 (8.6%) | 17 (0.8%) | 3 (8.6%) | 8 (22.9%) | 24 (68.6%) | 6.37 (1.2) |
| Venezuela | 43 (1.9%) | 32.24 (8.56) | 5.38 (4.55) | 19 (44.2%) | 23 (53.5%) | 16 (0.7%) | 9 (20.9%) | 23 (53.5%) | 11 (25.6%) | 5.52 (1.43) |
| Vietnam | 56 (2.5%) | 32.06 (7.99) | 16.6 (13.01) | 8 (14.3%) | 9 (16.1%) | 9 (0.4%) | 8 (14.3%) | 3 (5.4%) | 45 (80.4%) | 5.4 (1.8) |

Note. **^a^**The two columns show the number and percentage of participants from the given country that used HC in the past and in the present, respectively. So, for instance, if 25% women from Algeria used HC in the past, it means that 75% did not used HC in the past. Similarly, if 20.8% women used HC at the time of the study, it means that 79.2% did not usedHC at the time of the study. The third column shows the number and percentage of participants who either used HC in the past but at the time of the study not or did not use HC in the past but did use HC at the time of the study.

**Figure S1**

Distribution of participants who used hormonal contraception when meeting their romantic partners across countries.

**Figure S2**

Distribution of participants who currently use hormonal contraception across countries.

**Figure S3**

Distribution of participants who changed the pattern of hormonal contraception use across countries.

**Figure S4**

Distribution of participants who currently use any form of contraception across countries.

**Table S2**

Bivariate correlations across variables of interest.

| **Variable** | **1** | **2** | **3** | **4** | **5** | **6** | **7** | **8** | **9** |
| --- | --- | --- | --- | --- | --- | --- | --- | --- | --- |
| 1. Intimacy |  |  |  |  |  |  |  |  |  |
| 2. Passion | 0.841*** |  |  |  |  |  |  |  |  |
| 3. Commitment | 0.822*** | 0.791*** |  |  |  |  |  |  |  |
| 4. Relationship satisfaction | 0.799*** | 0.730*** | 0.718*** |  |  |  |  |  |  |
| 5. Congruence | 0.047* | 0.03 | 0.034 | 0.035 |  |  |  |  |  |
| 6. HC^a^ in the past | -0.008 | -0.01 | -0.014 | 0.005 | -0.146*** |  |  |  |  |
| 7. HC^a^ in the present | -0.015 | 0.01 | -0.021 | -0.014 | -0.340*** | 0.368*** |  |  |  |
| 8. Relationship length | -0.076*** | -0.123*** | -0.017 | -0.045* | -0.108*** | 0.094*** | -0.078*** |  |  |
| 9. Age | -0.079*** | -0.103*** | -0.046* | -0.060** | -0.044* | -0.005 | -0.028 | 0.366*** |  |
| 10. SES | 0.188*** | 0.192*** | 0.149*** | 0.214*** | 0.041 | -0.013 | -0.014 | 0.025 | 0.104*** |

*Note*. * *p* < .05, ** *p* < .01, *** *p* < .001. ^a^ Hormonal contraception.

**Table S3**

Results of multilevel models with relationship quality (i.e., love subscales and relationship satisfaction) as dependent variables and congruence of previous and current HC use as independent variables (not controlling for covariates).

| **Congruent HC use** | | | | | | | | |
| --- | --- | --- | --- | --- | --- | --- | --- | --- |
|  | **Intimacy** | | | | **Passion** | | | |
| **Fixed effects** | ***β*** | ***SE*** | ***95%CI*** | ***p*** | ***β*** | ***SE*** | ***95%CI*** | ***p*** |
| Congruent HC use | 0.048 | 0.021 | [0.007,0.090] | .024* | 0.035 | 0.021 | [-0.007,0.077] | .102 |
| **Random effects** | **Variance** | | **SD** | | **Variance** | | **SD** | |
| Intercept | 0.019 | | 0.139 | | 0.042 | | 0.205 | |
| Residual | 0.640 | | 0.800 | | 0.812 | | 0.901 | |
|  | **Commitment** | | | | **Relationship Satisfsaction** | | | |
| **Fixed effects** | ***β*** | ***SE*** | ***95%CI*** | ***p*** | ***β*** | ***SE*** | ***95%CI*** | ***p*** |
| Congruent HC use | -0.034 | 0.021 | [-0.008,0.076] | .116 | 0.036 | 0.021 | [-0.006,0.078] | .090 |
| **Random effects** | **Variance** | | **SD** | | **Variance** | | **SD** | |
| Intercept | 0.030 | | 0.173 | | 0.027 |  | 0.165 |  |
| Residual | 0.651 | | 0.807 | | 0.804 |  | 0.897 |  |
| **Current HC use** | | | | | | | | |
|  | **Intimacy** | | | | **Passion** | | | |
| **Fixed effects** | ***β*** | ***SE*** | ***95%CI*** | ***p*** | ***β*** | ***SE*** | ***95%CI*** | ***p*** |
| Current HC use | -0.023 | 0.022 | [-0.066,0.020] | .285 | -0.006 | 0.022 | [-0.049,0.037] | .797 |
| **Random effects** | **Variance** | | **SD** | | **Variance** | | **SD** | |
| Intercept | 0.020 | | 0.140 | | 0.042 | | 0.204 | |
| Residual | 0.641 | | 0.801 | | 0.813 | | 0.902 | |
|  | **Commitment** | | | | **Relationship Satisfsaction** | | | |
| **Fixed effects** | ***β*** | ***SE*** | ***95%CI*** | ***p*** | ***β*** | ***SE*** | ***95%CI*** | ***p*** |
| Current HC use | -0.025 | 0.022 | [-0.068,0.018] | .255 | -0.020 | 0.022 | [-0.063,0.023] | .359 |
| **Random effects** | **Variance** | | **SD** | | **Variance** | | **SD** | |
| Intercept | 0.030 | | 0.174 | | 0.027 |  | 0.166 |  |
| Residual | 0.651 | | 0.807 | | 0.805 |  | 0.897 |  |

*Note*. * *p* < .05, ** *p* < .01, *** *p* < .001. For Intimacy congruence model: ICC = 0.029, Pseudo *r^2^* fixed = 0.002, Pseudo *r^2^* total = 0.032, *df_residuals_* = 2220, deviance = 5361, for Passion congruence model: ICC = 0.049, Pseudo *r^2^* fixed = 0.001, Pseudo *r^2^*total = 0.050, *df_residuals_* = 2220, deviance = 5908.2, for Commitment congruence model: ICC = 0.044, Pseudo *r^2^* fixed = 0.001, Pseudo *r^2^* total = 0.045, *df_residuals_* = 2220, deviance = 5411.6, For Relationship Satisfaction congruence model: ICC = 0.033, Pseudo *r^2^* fixed = 0.001, Pseudo *r^2^* total = 0.034, *df_residuals_* = 2220, deviance = 5872, for Intimacy current use model: ICC = 0.030, Pseudo *r^2^* fixed = 0.001, Pseudo *r^2^* total = 0.030, *df_residuals_* = 2220, deviance = 5365, for Passion current use model: ICC = 0.049, Pseudo *r^2^* fixed = 0, Pseudo *r^2^*total = 0.049, *df_residuals_* = 2220, deviance = 5910.8, for Commitment current use model: ICC = 0.044, Pseudo *r^2^* fixed = 0.001, Pseudo *r^2^* total = 0.045, *df_residuals_* = 2220, deviance = 5412.8, For Relationship Satisfaction current use model: ICC = 0.033, Pseudo *r^2^* fixed = 0, Pseudo *r^2^* total = 0.033, *df_residuals_* = 2220, deviance = 5874.

**Table S4**

Results of multilevel models with relationship quality (i.e., love subscales and relationship satisfaction) as dependent variables and congruence of previous and current HC use as independent variables, controlling for covariates.

| **Congruent HC use** | | | | | | | | |
| --- | --- | --- | --- | --- | --- | --- | --- | --- |
|  | **Intimacy** | | | | **Passion** | | | |
| **Fixed effects** | **β** | **SE** | **95%CI** | **p** | **β** | **SE** | **95%CI** | **p** |
| Congruent HC use | 0.035 | 0.021 | [-0.006,0.076] | 0.096 | 0.017 | 0.021 | [-0.024,0.058] | 0.424 |
| Relationship length | -0.041 | 0.024 | [-0.087,0.005] | 0.081 | -0.078 | 0.024 | [-0.124,-0.031] | <.001*** |
| Age | -0.085 | 0.023 | [-0.129,-0.04] | <.001*** | -0.1 | 0.023 | [-0.144,-0.055] | <.001*** |
| SES | 0.188 | 0.021 | [0.146,0.23] | <.001*** | 0.185 | 0.021 | [0.144,0.227] | <.001*** |
| **Random effects** | **Variance** | | **SD** | | **Variance** | | **SD** | |
| Intercept | 0.014 | | 0.118 | | 0.030 | | 0.172 | |
| Residual | 0.615 | | 0.784 | | 0.775 | | 0.88 | |
|  | **Commitment** | | | | **Relationship Satisfaction** | | | |
| **Fixed effects** | **β** | **SE** | **95%CI** | **p** | **β** | **SE** | **95%CI** | **p** |
| Congruent HC use | 0.03 | 0.021 | [-0.012,0.071] | 0.164 | 0.026 | 0.021 | [-0.015,0.067] | 0.216 |
| Relationship length | 0.017 | 0.024 | [-0.03,0.064] | 0.48 | -0.014 | 0.024 | [-0.06,0.032] | 0.56 |
| Age | -0.074 | 0.023 | [-0.119,-0.029] | <.001*** | -0.078 | 0.023 | [-0.122,-0.033] | <.001*** |
| SES | 0.137 | 0.022 | [0.095,0.179] | <.001*** | 0.21 | 0.021 | [0.169,0.252] | <.001*** |
| **Random effects** | **Variance** | | **SD** | | **Variance** | | **SD** | |
| Intercept | 0.026 | | 0.161 | | 0.018 | | 0.134 | |
| Residual | 0.638 | | 0.799 | | 0.771 | | 0.878 | |
| **Current HC use** | | | | | | | | |
|  | **Intimacy** | | | | **Passion** | | | |
| **Fixed effects** | **β** | **SE** | **95%CI** | **p** | **β** | **SE** | **95%CI** | **p** |
| Current HC use | -0.029 | 0.021 | [-0.07,0.013] | 0.183 | -0.013 | 0.021 | [-0.055,0.029] | 0.546 |
| Relationship length | -0.047 | 0.023 | [-0.093,-0.001] | 0.047* | -0.08 | 0.024 | [-0.127,-0.034] | <.001*** |
| Age | -0.085 | 0.023 | [-0.13,-0.041] | <.001*** | -0.1 | 0.023 | [-0.144,-0.056] | <.001*** |
| SES | 0.189 | 0.021 | [0.148,0.231] | <.001*** | 0.186 | 0.021 | [0.144,0.227] | <.001*** |
| Random effects | Variance | | SD | | Variance | | SD | |
| Intercept | 0.014 | | 0.119 | | 0.03 | | 0.172 | |
| Residual | 0.615 | | 0.785 | | 0.775 | | 0.88 | |
|  | **Commitment** | | | | **Relationship Satisfaction** | | | |
| **Fixed effects** | **β** | **SE** | **95%CI** | **p** | **β** | **SE** | **95%CI** | **p** |
| Current HC use | -0.028 | 0.022 | [-0.071,0.014] | 0.192 | -0.024 | 0.021 | [-0.066,0.018] | 0.264 |
| Relationship length | 0.012 | 0.024 | [-0.035,0.059] | 0.610 | -0.018 | 0.023 | [-0.064,0.028] | 0.442 |
| Age | -0.074 | 0.023 | [-0.119,-0.029] | <.001*** | -0.078 | 0.023 | [-0.122,-0.034] | <.001*** |
| SES | 0.138 | 0.022 | [0.096,0.18] | <.001*** | 0.211 | 0.021 | [0.17,0.253] | <.001*** |
| **Random effects** | **Variance** | | **SD** | | **Variance** | | **SD** | |
| Intercept | 0.026 | | 0.161 | | 0.018 | | 0.134 | |
| Residual | 0.638 | | 0.799 | | 0.771 | | 0.878 | |

*Note*. * *p* < .05, ** *p* < .01, *** *p* < .001. For Intimacy congruence model: ICC = 0.022, Pseudo *r^2^* fixed = 0.046, Pseudo *r^2^* total = 0.067, *df_residuals_* = 2217, deviance = 5266.2, for Passion congruence model: ICC = 0.037, Pseudo *r^2^* fixed = 0.053, Pseudo *r^2^* total = 0.088, *df_residuals_* = 2217, deviance = 5794.5, for Commitment congruence model: ICC = 0.039, Pseudo *r^2^* fixed = 0.023, Pseudo *r^2^* total = 0.061, *df_residuals_* = 2217, deviance = 5364, for Relationship Satisfaction congruence model: ICC = 0.023, Pseudo *r^2^* fixed = 0.049, Pseudo *r^2^* total = 0.071, df_residuals_ = 2217, deviance = 5767.3, for Intimacy current use model: ICC = 0.023, Pseudo *r^2^* fixed = 0.045, Pseudo *r^2^* total = 0.067, *df_residuals_* = 2217, deviance = 5267.2, for Passion current use model: ICC = 0.037, Pseudo *r^2^* fixed = 0.053, Pseudo *r^2^* total = 0.088, *df_residuals_* = 2217, deviance = 5794.8, for Commitment current use model: ICC = 0.039, Pseudo *r^2^* fixed = 0.023, Pseudo *r^2^* total = 0.061, *df_residuals_* = 2217, deviance = 5364.2, for Relationship Satisfaction current use model: ICC = 0.023, Pseudo *r^2^* = .049, *r^2^* total = 0.071, *df_residuals_* = 2217, deviance = 5767.6.

**Figure S5**

Love intensity across participants who used HCs in the past (or not) and who now use HCs (or not), controlling for participants’ age, relationship length, and SES.

*Note*. Error bars represent 95% confidence intervals. Importantly although visually, the 95% confidence intervals for the congruent groups (the 'Yes/Yes' and 'No/No' clusters) appear higher than the incongruent groups, the formal statistical tests for the interaction were non-significant. First, Figure displays weighted means and confidence intervals. In contrast, our reported results are based on Linear Mixed Models that adjust for several significant predictors, while accounting for country-level variance as a random effect. Second, a significant interaction requires that the effect of current HC use differs significantly depending on past HC use. In our data, while quality is numerically higher in congruent states, the “drop” in quality associated with being incongruent was consistent across both past-user conditions. Mathematically, this results in a non-significant interaction term, even if individual group means appear distinct. Third, while non-overlapping 95% CIs between two groups could suggest a significant pairwise difference, they do not guarantee a significant interaction in a complex factorial design, especially when the model accounts for nesting (Country) and multiple continuous covariates that the figure does not visualize.

**Table S5**

Results of multilevel models with relationship quality (i.e., love subscales and relationship satisfaction) as dependent variables and congruence of HC use and current HC use as independent variables, controlling for covariates and weighted propensity scores.

| **Congruent HC use** | | | | | | | | | | | | |
| --- | --- | --- | --- | --- | --- | --- | --- | --- | --- | --- | --- | --- |
|  | **Intimacy** | | | | | | **Passion** | | | | | |
| **Fixed effects** | ***β*** | ***SE*** | ***95%CI*** | | ***p*** | ***β*** | | ***SE*** | ***95%CI*** | | ***p*** |  |
| Congruent HC use | -0.003 | 0.021 | [-0.045, 0.038] | | 0.870 | -0.042 | | 0.021 | [-0.083, 0.000] | | 0.049* |  |
| Relationship length | -0.036 | 0.023 | [-0.082, 0.010] | | 0.127 | -0.077 | | 0.023 | [-0.123, -0.032] | | <.001*** |  |
| Age | -0.086 | 0.023 | [-0.130, -0.041] | | <.001*** | -0.094 | | 0.023 | [-0.138, -0.050] | | <.001*** |  |
| SES | 0.18 | 0.021 | [ 0.138, 0.222] | | <.001*** | 0.166 | | 0.021 | [ 0.124, 0.207] | | <.001*** |  |
| **Random effects** | **Variance** | | | **SD** | | | **Variance** | | | **SD** | | |
| Intercept | 0.025 | | | 0.157 | | | 0.037 | | | 0.192 | | |
| Residual | 0.564 | | | 0.751 | | | 0.716 | | | 0.846 | | |
|  | **Commitment** | | | | | | **Relationship Satisfaction** | | | | | |
| **Fixed effects** | ***β*** | ***SE*** | ***95%CI*** | | ***p*** | ***β*** | | ***SE*** | ***95%CI*** | | ***p*** |  |
| Congruent HC use | -0.011 | 0.021 | [-0.053, 0.031] | | 0.618 | -0.009 | | 0.021 | [-0.051, 0.032] | | 0.656 |  |
| Relationship length | 0.005 | 0.024 | [-0.041, 0.052] | | 0.826 | -0.006 | | 0.023 | [-0.052, 0.039] | | 0.784 |  |
| Age | -0.07 | 0.023 | [-0.116, -0.025] | | 0.002** | -0.074 | | 0.023 | [-0.119, -0.029] | | <.001*** |  |
| SES | 0.124 | 0.022 | [ 0.082, 0.167] | | <.001*** | 0.2 | | 0.021 | [ 0.158, 0.242] | | <.001*** |  |
| **Random effects** | **Variance** | | | **SD** | | | **Variance** | | | **SD** | | |
| Intercept | 0.034 | | | 0.185 | | | 0.033 | | | 0.181 | | |
| Residual | 0.583 | | | 0.764 | | | 0.733 | | | 0.856 | | |
| **Current HC use** | | | | | | | | | | | | |
|  | **Intimacy** | | | | | | **Passion** | | | | | |
| **Fixed effects** | ***β*** | ***SE*** | ***95%CI*** | | ***p*** | ***β*** | | ***SE*** | ***95%CI*** | | ***p*** |  |
| Current HC use | -0.033 | 0.022 | [-0.076, 0.009] | | 0.125 | 2.43E-04 | | 0.022 | [-0.042, 0.043] | | 0.991 |  |
| Relationship length | -0.037 | 0.023 | [-0.083, 0.009] | | 0.115 | -0.076 | | 0.023 | [-0.121, -0.030] | | <.001*** |  |
| Age | -0.086 | 0.023 | [-0.131, -0.042] | | <.001*** | -0.094 | | 0.023 | [-0.138, -0.050] | | <.001*** |  |
| SES | 0.18 | 0.021 | [ 0.138, 0.222] | | <.001*** | 0.165 | | 0.021 | [ 0.124, 0.207] | | <.001*** |  |
| **Random effects** | **Variance** | | | **SD** | | | **Variance** | | | **SD** | | |
| Intercept | 0.026 | | | 0.161 | | | 0.039 | | | 0.197 | | |
| Residual | 0.563 | | | 0.750 | | | 0.716 | | | 0.846 | | |
|  | **Commitment** | | | | | | **Relationship Satisfaction** | | | | | |
| **Fixed effects** | ***β*** | ***SE*** | ***95%CI*** | | ***p*** | ***β*** | | ***SE*** | ***95%CI*** | | ***p*** |  |
| Current HC use | -0.022 | 0.022 | [-0.066, 0.021] | | 0.307 | -0.026 | | 0.022 | [-0.068, 0.017] | | 0.235 |  |
| Relationship length | 0.005 | 0.024 | [-0.042, 0.051] | | 0.844 | -0.007 | | 0.023 | [-0.053, 0.039] | | 0.76 |  |
| Age | -0.071 | 0.023 | [-0.116, -0.026] | | 0.002** | -0.075 | | 0.023 | [-0.119, -0.030] | | <.001*** |  |
| SES | 0.124 | 0.022 | [ 0.082, 0.167] | | <.001*** | 0.2 | | 0.021 | [ 0.158, 0.242] | | <.001*** |  |
| **Random effects** | **Variance** | | | **SD** | | | **Variance** | | | **SD** | | |
| Intercept | 0.035 | | | 0.188 | | | 0.034 | | | 0.185 | | |
| Residual | 0.583 | | | 0.764 | | | 0.732 | | | 0.856 | | |

*Note*. * *p* < .05, ** *p* < .01, *** *p* < .001. For Intimacy congruence model: ICC = 0.042, Pseudo *r^2^* fixed = 0.040, Pseudo *r^2^* total = 0.080, *df_residuals_* = 2217, deviance = 5418.7, for Passion congruence model: ICC = 0.049, Pseudo *r^2^* fixed = 0.045, Pseudo *r^2^* total = 0.092, *df_residuals_* = 2217, deviance = 5955.2, for Commitment congruence model: ICC = 0.055, Pseudo *r^2^* fixed = 0.018, Pseudo *r^2^* total = 0.073, *df_residuals_* = 2217, deviance = 5505.9, for Relationship Satisfaction congruence model: ICC = 0.043, Pseudo *r^2^* fixed = 0.043, Pseudo *r^2^* total = 0.084, *df_residuals_* = 2217, deviance = 6003.3, for Intimacy current use model: ICC = 0.044, Pseudo *r^2^* fixed = 0.041, Pseudo *r^2^* total = 0.084, *df_residuals_* = 2217, deviance = 5416.4, for Passion current use model: ICC = 0.051, Pseudo *r^2^* fixed = 0.045, Pseudo *r^2^* total = 0.094, *df_residuals_* = 2217, deviance = 5959.1, for Commitment current use model: ICC = 0.057, Pseudo *r^2^* fixed = 0.019, Pseudo *r^2^* total = 0.075, *df_residuals_* = 2217, deviance = 5505.2, for Relationship Satisfaction current use model: ICC = 0.045, Pseudo *r^2^* fixed = 0.044, Pseudo *r^2^* total = 0.086, *df_residuals_* = 2217, deviance = 6002.1.

**Table S6**

Results of multilevel models with relationship quality (i.e., love subscales and relationship satisfaction) as dependent variables and congruence of previous and current HC use as independent variables, controlling for covariates on a subsample of heterosexual women (*N* = 1,808).

| **Congruent HC use** | | | | | | | | |
| --- | --- | --- | --- | --- | --- | --- | --- | --- |
|  | **Intimacy** | | | | **Passion** | | | |
| **Fixed effects** | **β** | **SE** | **95%CI** | **p** | **β** | **SE** | **95%CI** | **p** |
| Congruent HC use | 0.027 | 0.023 | [-0.018,0.073] | 0.242 | 0.011 | 0.023 | [-0.035,0.056] | 0.638 |
| Relationship length | -0.031 | 0.026 | [-0.082,0.02] | 0.231 | -0.071 | 0.026 | [-0.122,-0.02] | 0.006** |
| Age | -0.107 | 0.025 | [-0.156,-0.058] | <.001*** | -0.118 | 0.025 | [-0.166,-0.069] | <.001*** |
| SES | 0.19 | 0.024 | [0.144,0.236] | <.001*** | 0.194 | 0.023 | [0.148,0.24] | <.001*** |
| **Random effects** | **Variance** | | **SD** | | **Variance** | | **SD** | |
| Intercept | 0.014 | | 0.12 | | 0.028 | | 0.168 | |
| Residual | 0.625 | | 0.791 | | 0.783 | | 0.885 | |
|  | **Commitment** | | | | **Relationship Satisfaction** | | | |
| **Fixed effects** | **β** | **SE** | **95%CI** | **p** | **β** | **SE** | **95%CI** | **p** |
| Congruent HC use | 0.028 | 0.024 | [-0.018,0.074] | 0.233 | 0.019 | 0.023 | [-0.027,0.065] | 0.413 |
| Relationship length | 0.023 | 0.027 | [-0.03,0.075] | 0.395 | 0.002 | 0.026 | [-0.049,0.053] | 0.937 |
| Age | -0.089 | 0.025 | [-0.138,-0.039] | <.001*** | -0.101 | 0.025 | [-0.15,-0.052] | <.001*** |
| SES | 0.145 | 0.024 | [0.098,0.192] | <.001*** | 0.21 | 0.023 | [0.163,0.256] | <.001*** |
| **Random effects** | **Variance** | | **SD** | | **Variance** | | **SD** | |
| Intercept | 0.025 | | 0.157 | | 0.02 | | 0.143 | |
| Residual | 0.649 | | 0.806 | | 0.764 | | 0.874 | |
| **Current HC use** | | | | | | | | |
|  | **Intimacy** | | | | **Passion** | | | |
| **Fixed effects** | **β** | **SE** | **95%CI** | **p** | **β** | **SE** | **95%CI** | **p** |
| Current HC use | 0.008 | 0.024 | [-0.039,0.055] | 0.737 | 0.022 | 0.024 | [-0.024,0.069] | 0.345 |
| Relationship length | -0.034 | 0.026 | [-0.085,0.016] | 0.184 | -0.072 | 0.026 | [-0.123,-0.021] | 0.006** |
| Age | -0.107 | 0.025 | [-0.156,-0.058] | <.001*** | -0.117 | 0.025 | [-0.165,-0.068] | <.001*** |
| SES | 0.191 | 0.024 | [0.145,0.237] | <.001*** | 0.194 | 0.023 | [0.149,0.24] | <.001*** |
| Random effects | Variance | | SD | | Variance | | SD | |
| Intercept | 0.013 | | 0.116 | | 0.026 | | 0.162 | |
| Residual | 0.626 | | 0.791 | | 0.783 | | 0.885 | |
|  | **Commitment** | | | | **Relationship Satisfaction** | | | |
| **Fixed effects** | **β** | **SE** | **95%CI** | **p** | **β** | **SE** | **95%CI** | **p** |
| Current HC use | 0.004 | 0.024 | [-0.043,0.052] | 0.86 | 0.013 | 0.024 | [-0.033,0.06] | 0.572 |
| Relationship length | 0.019 | 0.026 | [-0.033,0.071] | 0.474 | 0 | 0.026 | [-0.051,0.051] | 0.998 |
| Age | -0.088 | 0.025 | [-0.138,-0.038] | <.001*** | -0.101 | 0.025 | [-0.15,-0.052] | <.001*** |
| SES | 0.146 | 0.024 | [0.099,0.192] | <.001*** | 0.21 | 0.023 | [0.164,0.256] | <.001*** |
| **Random effects** | **Variance** | | **SD** | | **Variance** | | **SD** | |
| Intercept | 0.024 | | 0.155 | | 0.019 | | 0.139 | |
| Residual | 0.650 | | 0.806 | | 0.764 | | 0.874 | |

*Note*. * *p* < .05, ** *p* < .01, *** *p* < .001. For Intimacy congruence model: ICC = 0.022, Pseudo *r^2^* fixed = 0.048, Pseudo *r^2^* total = 0.070, *df_residuals_* = 1801, deviance = 4311.6, for Passion congruence model: ICC = 0.035, Pseudo *r^2^* fixed = 0.058, Pseudo *r^2^* total = 0.091, *df_residuals_* = 1801, deviance = 4728.9, for Commitment congruence model: ICC = 0.037, Pseudo *r^2^* fixed = 0.027, Pseudo *r^2^* total = 0.063, *df_residuals_* = 1801, deviance = 4393.3, for Relationship Satisfaction congruence model: ICC = 0.026, Pseudo *r^2^* fixed = 0.051, Pseudo *r^2^* total = 0.076, df_residuals_ = 1801, deviance = 4676.4, for Intimacy current use model: ICC = 0.021, Pseudo *r^2^* fixed = 0.048, Pseudo *r^2^* total = 0.068, *df_residuals_* = 1801, deviance = 4312.9, for Passion current use model: ICC = 0.033, Pseudo *r^2^* fixed = 0.059, Pseudo *r^2^* total = 0.089, *df_residuals_* = 1801, deviance = 4728.3, for Commitment current use model: ICC = 0.035, Pseudo *r^2^* fixed = 0.026, Pseudo *r^2^* total = 0.060, *df_residuals_* = 1801, deviance = 4394.7, for Relationship Satisfaction current use model: ICC = 0.025, Pseudo *r^2^* = .051, *r^2^* total = 0.074, *df_residuals_* = 1801, deviance = 4676.7.

**Table S7**

Results of multilevel models with relationship quality (i.e., love subscales and relationship satisfaction) as dependent variables and congruence of previous and current HC use as independent variables, controlling for covariates on a subsample excluding data from Japan and India (*N* = 2,148).

| **Congruent HC use** | | | | | | | | |
| --- | --- | --- | --- | --- | --- | --- | --- | --- |
|  | **Intimacy** | | | | **Passion** | | | |
| **Fixed effects** | **β** | **SE** | **95%CI** | **p** | **β** | **SE** | **95%CI** | **p** |
| Congruent HC use | 0.038 | 0.021 | [-0.004,0.08] | 0.075 | 0.019 | 0.021 | [-0.022,0.061] | 0.365 |
| Relationship length | -0.043 | 0.024 | [-0.09,0.004] | 0.074 | -0.073 | 0.024 | [-0.12,-0.026] | 0.003** |
| Age | -0.091 | 0.023 | [-0.137,-0.046] | <.001*** | -0.113 | 0.023 | [-0.158,-0.068] | <.001*** |
| SES | 0.182 | 0.022 | [0.14,0.225] | <.001*** | 0.187 | 0.021 | [0.145,0.229] | <.001*** |
| **Random effects** | **Variance** | | **SD** | | **Variance** | | **SD** | |
| Intercept | 0.015 | | 0.123 | | 0.027 | | 0.165 | |
| Residual | 0.613 | | 0.783 | | 0.774 | | 0.879 | |
|  | **Commitment** | | | | **Relationship Satisfaction** | | | |
| **Fixed effects** | **β** | **SE** | **95%CI** | **p** | **β** | **SE** | **95%CI** | **p** |
| Congruent HC use | 0.035 | 0.022 | [-0.007,0.078] | 0.104 | 0.029 | 0.021 | [-0.013,0.07] | 0.182 |
| Relationship length | 0.022 | 0.025 | [-0.026,0.07] | 0.367 | -0.012 | 0.024 | [-0.059,0.035] | 0.621 |
| Age | -0.083 | 0.023 | [-0.129,-0.037] | <.001*** | -0.087 | 0.023 | [-0.132,-0.042] | <.001*** |
| SES | 0.135 | 0.022 | [0.093,0.178] | <.001*** | 0.208 | 0.022 | [0.166,0.251] | <.001*** |
| **Random effects** | **Variance** | | **SD** | | **Variance** | | **SD** | |
| Intercept | 0.022 | | 0.149 | | 0.768 | | 0.876 | |
| Residual | 0.636 | | 0.798 | | 0.018 | | 0.134 | |
| **Current HC use** | | | | | | | | |
|  | **Intimacy** | | | | **Passion** | | | |
| **Fixed effects** | **β** | **SE** | **95%CI** | **p** | **β** | **SE** | **95%CI** | **p** |
| Current HC use | -0.029 | 0.022 | [-0.072,0.014] | 0.181 | -0.012 | 0.022 | [-0.055,0.03] | 0.572 |
| Relationship length | -0.049 | 0.024 | [-0.096,-0.002] | 0.04* | -0.076 | 0.024 | [-0.123,-0.029] | 0.002** |
| Age | -0.092 | 0.023 | [-0.137,-0.047] | <.001*** | -0.113 | 0.023 | [-0.158,-0.068] | <.001*** |
| SES | 0.184 | 0.022 | [0.141,0.226] | <.001*** | 0.188 | 0.021 | [0.146,0.23] | <.001*** |
| Random effects | Variance | | SD | | Variance | | SD | |
| Intercept | 0.015 | | 0.124 | | 0.027 | | 0.165 | |
| Residual | 0.613 | | 0.783 | | 0.774 | | 0.88 | |
|  | **Commitment** | | | | **Relationship Satisfaction** | | | |
| **Fixed effects** | **β** | **SE** | **95%CI** | **p** | **β** | **SE** | **95%CI** | **p** |
| Current HC use | -0.029 | 0.022 | [-0.072,0.014] | 0.188 | -0.023 | 0.022 | [-0.066,0.019] | 0.284 |
| Relationship length | 0.016 | 0.024 | [-0.032,0.064] | 0.502 | -0.017 | 0.024 | [-0.063,0.03] | 0.488 |
| Age | -0.084 | 0.023 | [-0.129,-0.038] | <.001*** | -0.087 | 0.023 | [-0.132,-0.042] | <.001*** |
| SES | 0.137 | 0.022 | [0.094,0.179] | <.001*** | 0.209 | 0.022 | [0.167,0.252] | <.001*** |
| **Random effects** | **Variance** | | **SD** | | **Variance** | | **SD** | |
| Intercept | 0.022 | | 0.149 | | 0.018 | | 0.134 | |
| Residual | 0.636 | | 0.798 | | 0.768 | | 0.877 | |

*Note*. * *p* < .05, ** *p* < .01, *** *p* < .001. For Intimacy congruence model: ICC = 0.024, Pseudo *r^2^* fixed = 0.046, Pseudo *r^2^* total = 0.068, *df_residuals_* = 2141, deviance = 5080.1, for Passion congruence model: ICC = 0.034, Pseudo *r^2^* fixed = 0.056, Pseudo *r^2^* total = 0.088, *df_residuals_* = 2141, deviance = 5589.4, for Commitment congruence model: ICC = 0.034, Pseudo *r^2^* fixed = 0.024, Pseudo *r^2^* total = 0.057, *df_residuals_* = 2141, deviance = 5169.4, for Relationship Satisfaction congruence model: ICC = 0.023, Pseudo *r^2^* fixed = 0.050, Pseudo *r^2^* total = 0.072, df_residuals_ = 2141, deviance = 5563.4, for Intimacy current use model: ICC = 0.024, Pseudo *r^2^* fixed = 0.045, Pseudo *r^2^* total = 0.068, *df_residuals_* = 2141, deviance = 5081.5, for Passion current use model: ICC = 0.034, Pseudo *r^2^* fixed = 0.056, Pseudo *r^2^* total = 0.088, *df_residuals_* = 2141, deviance = 5589.9, for Commitment current use model: ICC = 0.034, Pseudo *r^2^* fixed = 0.024, Pseudo *r^2^* total = 0.057, *df_residuals_* = 2141, deviance = 5170.3, for Relationship Satisfaction current use model: ICC = 0.023, Pseudo *r^2^* = .050, *r^2^* total = 0.072, *df_residuals_* = 2141, deviance = 5564.1.

**Table S8**

Results of multilevel models with relationship quality (i.e., love subscales and relationship satisfaction) as dependent variables and congruence of previous and current HC use as independent variables, along with an interaction term between countries’ cultural distance from the USA (a measure of WEIRDedness), controlling for covariates.

| **Congruent HC use** | | | | | | | | |
| --- | --- | --- | --- | --- | --- | --- | --- | --- |
|  | **Intimacy** | | | | **Passion** | | | |
| **Fixed effects** | **β** | **SE** | **95%CI** | **p** | **β** | **SE** | **95%CI** | **p** |
| Congruent HC use | 0.048 | 0.025 | [-0.001,0.097] | 0.054 | 0.027 | 0.025 | [-0.022,0.076] | 0.284 |
| Cultural distance | 0.042 | 0.033 | [-0.022,0.105] | 0.199 | 0.06 | 0.037 | [-0.012,0.132] | 0.101 |
| Relationship length | -0.029 | 0.029 | [-0.085,0.027] | 0.303 | -0.053 | 0.029 | [-0.109,0.003] | 0.066 |
| Age | -0.124 | 0.027 | [-0.177,-0.071] | <.001*** | -0.14 | 0.027 | [-0.193,-0.087] | <.001*** |
| SES | 0.211 | 0.025 | [0.161,0.261] | <.001*** | 0.205 | 0.025 | [0.155,0.255] | <.001*** |
| Congruent HC use x Cultural distance | 0.018 | 0.025 | [-0.03,0.066] | 0.459 | 0.019 | 0.024 | [-0.029,0.067] | 0.437 |
| **Random effects** | **Variance** | | **SD** | | **Variance** | | **SD** | |
| Intercept | 0.008 | | 0.091 | | 0.019 | | 0.14 | |
| Residual | 0.609 | | 0.781 | | 0.772 | | 0.879 | |
|  | **Commitment** | | | | **Relationship Satisfaction** | | | |
| **Fixed effects** | **β** | **SE** | **95%CI** | **p** | **β** | **SE** | **95%CI** | **p** |
| Congruent HC use | 0.033 | 0.025 | [-0.017,0.083] | 0.191 | 0.042 | 0.025 | [-0.007,0.091] | 0.095 |
| Cultural distance | 0.057 | 0.038 | [-0.018,0.131] | 0.138 | 0.027 | 0.036 | [-0.044,0.098] | 0.455 |
| Relationship length | 0.041 | 0.029 | [-0.016,0.099] | 0.16 | -0.007 | 0.029 | [-0.064,0.05] | 0.805 |
| Age | -0.1 | 0.028 | [-0.154,-0.046] | <.001*** | -0.097 | 0.027 | [-0.15,-0.043] | <.001*** |
| SES | 0.168 | 0.026 | [0.117,0.219] | <.001*** | 0.221 | 0.026 | [0.171,0.271] | <.001*** |
| Congruent HC use x Cultural distance | 0 | 0.025 | [-0.049,0.048] | 0.989 | 0.013 | 0.025 | [-0.035,0.061] | 0.591 |
| **Random effects** | **Variance** | | **SD** | | **Variance** | | **SD** | |
| Intercept | 0.017 | | 0.13 | | 0.018 | | 0.133 | |
| Residual | 0.606 | | 0.778 | | 0.767 | | 0.876 | |
| **Current HC use** | | | | | | | | |
|  | **Intimacy** | | | | **Passion** | | | |
| **Fixed effects** | **β** | **SE** | **95%CI** | **p** | **β** | **SE** | **95%CI** | **p** |
| Current HC use | -0.024 | 0.025 | [-0.073,0.025] | 0.332 | -0.018 | 0.025 | [-0.067,0.031] | 0.474 |
| Cultural distance | 0.047 | 0.032 | [-0.015,0.11] | 0.139 | 0.063 | 0.036 | [-0.008,0.135] | 0.083 |
| Relationship length | -0.033 | 0.028 | [-0.088,0.023] | 0.245 | -0.054 | 0.028 | [-0.109,0.002] | 0.058 |
| Age | -0.124 | 0.027 | [-0.177,-0.07] | <.001*** | -0.141 | 0.027 | [-0.194,-0.088] | <.001*** |
| SES | 0.213 | 0.025 | [0.163,0.263] | <.001*** | 0.206 | 0.025 | [0.156,0.256] | <.001*** |
| Congruent HC use x Cultural distance | -0.029 | 0.025 | [-0.077,0.019] | 0.239 | -0.015 | 0.025 | [-0.063,0.034] | 0.546 |
| Random effects | Variance | | SD | | Variance | | SD | |
| Intercept | 0.008 | | 0.087 | | 0.019 | | 0.139 | |
| Residual | 0.61 | | 0.781 | | 0.772 | | 0.879 | |
|  | **Commitment** | | | | **Relationship Satisfaction** | | | |
| **Fixed effects** | **β** | **SE** | **95%CI** | **p** | **β** | **SE** | **95%CI** | **p** |
| Current HC use | -0.024 | 0.026 | [-0.074,0.026] | 0.346 | -0.023 | 0.025 | [-0.073,0.026] | 0.358 |
| Cultural distance | 0.061 | 0.038 | [-0.013,0.134] | 0.107 | 0.03 | 0.036 | [-0.041,0.102] | 0.404 |
| Relationship length | 0.037 | 0.029 | [-0.02,0.093] | 0.206 | -0.012 | 0.029 | [-0.068,0.044] | 0.676 |
| Age | -0.098 | 0.028 | [-0.152,-0.044] | <.001*** | -0.099 | 0.027 | [-0.152,-0.045] | <.001*** |
| SES | 0.17 | 0.026 | [0.12,0.221] | <.001*** | 0.221 | 0.026 | [0.171,0.272] | <.001*** |
| Congruent HC use x Cultural distance | -0.027 | 0.025 | [-0.077,0.022] | 0.278 | 0.006 | 0.025 | [-0.043,0.055] | 0.819 |
| **Random effects** | **Variance** | | **SD** | | **Variance** | | **SD** | |
| Intercept | 0.016 | | 0.126 | | 0.018 | | 0.135 | |
| Residual | 0.606 | | 0.778 | | 0.768 | | 0.877 | |

*Note*. * *p* < .05, ** *p* < .01, *** *p* < .001. For Intimacy congruence model: ICC = 0.013, Pseudo *r^2^* fixed = 0.065, Pseudo *r^2^* total = 0.078, *df_residuals_* = 1548, deviance = 3663.3, for Passion congruence model: ICC = 0.025, Pseudo *r^2^* fixed = 0.072, Pseudo *r^2^* total = 0.095, *df_residuals_* = 1548, deviance = 4041.4, for Commitment congruence model: ICC = 0.027, Pseudo *r^2^* fixed = 0.039, Pseudo *r^2^* total = 0.065, *df_residuals_* = 1548, deviance = 3665.5, for Relationship Satisfaction congruence model: ICC = 0.023, Pseudo *r^2^* fixed = 0.059, Pseudo *r^2^* total = 0.080, df_residuals_ = 1548, deviance = 4031, for Intimacy current use model: ICC = 0.012, Pseudo *r^2^* fixed = 0.064, Pseudo *r^2^* total = 0.076, *df_residuals_* = 1548, deviance = 3665, for Passion current use model: ICC = 0.024, Pseudo *r^2^* fixed = 0.072, Pseudo *r^2^* total = 0.094, *df_residuals_* = 1548, deviance 4042.1, for Commitment current use model: ICC = 0.025, Pseudo *r^2^* fixed = 0.039, Pseudo *r^2^* total = 0.064, *df_residuals_* = 1548, deviance = 3665.1, for Relationship Satisfaction current use model: ICC = 0.023, Pseudo *r^2^* = .058, *r^2^* total = 0.080, *df_residuals_* = 1548, deviance = 4033.1.
